# α-Tomatine-Mediated Anti-Cancer Activity *In Vitro* and *In Vivo* through Cell Cycle- and Caspase-Independent Pathways

**DOI:** 10.1371/journal.pone.0044093

**Published:** 2012-09-06

**Authors:** Min-Wu Chao, Chun-Han Chen, Ya-Ling Chang, Che-Ming Teng, Shiow-Lin Pan

**Affiliations:** 1 Phamacological Institute, College of Medicine, National Taiwan University, Taipei, Taiwan; 2 Department of Biotechnology and Pharmaceutical Research, National Health Research Institutes, Miaoli County, Taiwan; University of Pecs Medical School, Hungary

## Abstract

α-Tomatine, a tomato glycoalkaloid, has been reported to possess antibiotic properties against human pathogens. However, the mechanism of its action against leukemia remains unclear. In this study, the therapeutic potential of α-tomatine against leukemic cells was evaluated *in vitro* and *in vivo*. Cell viability experiments showed that α-tomatine had significant cytotoxic effects on the human leukemia cancer cell lines HL60 and K562, and the cells were found to be in the Annexin V-positive/propidium iodide-negative phase of cell death. In addition, α-tomatine induced both HL60 and K562 cell apoptosis in a cell cycle- and caspase-independent manner. α-Tomatine exposure led to a loss of the mitochrondrial membrane potential, and this finding was consistent with that observed on activation of the Bak and Mcl-1 short form (Mcl-1s) proteins. Exposure to α-tomatine also triggered the release of the apoptosis-inducing factor (AIF) from the mitochondria into the nucleus and down-regulated survivin expression. Furthermore, α-tomatine significantly inhibited HL60 xenograft tumor growth without causing loss of body weight in severe combined immunodeficiency (SCID) mice. Immunohistochemical test showed that the reduced tumor growth in the α-tomatine-treated mice was a result of increased apoptosis, which was associated with increased translocation of AIF in the nucleus and decreased survivin expression *ex vivo*. These results suggest that α-tomatine may be a candidate for leukemia treatment.

## Introduction

α-Tomatine is a glycoalkaloid found in the tomato (*Lycopersicon esculentum*). It was first isolated by Fontaine *et al*, and was found to be abundant in immature green tomatoes (500 mg α-tomatine/kg of fresh fruit) [1]; however, it is largely degraded as tomatoes ripen (5 mg/kg) [2]. The antibiotic and immunological effects of α-tomatine have been reported [3], [4]. α-Tomatine has also exhibited anti-proliferative and apoptotic activity through inactivation of the phosphoinostide 3-kinase (PI3K)/AKT pathway or nuclear factor (NF)-κB activation in solid tumor cell lines such as liver, colon, and lung cancer cells [1], [5], [6]. However, the role of α-tomatine in leukemic cells remains unknown.

Leukemia, which is the common hematological neoplasm, it is a malignancy that affects blood precursor cells in the bone marrow. Immature or incompletely differentiated blood cells accumulate in the bone marrow and replace normal blood cells. In recent years, the incidence and mortality rates for leukemia have increased and chemotherapy has been the major strategy adopted for the treatment and cure of leukemia. However, the most definitive and effective treatment for leukemia has been bone marrow transplantation. Nevertheless, due to the high recurrence rate of leukemia, low success ratio of bone marrow transplantation, lack of highly selective chemotherapy options, and serious adverse effects of both treatment approaches, investigators continue to search for and develop drugs that are more selective and less toxic for effective treatment of leukemia [7].

Apoptosis is a type of programmed cell death and the apoptotic cascade can be initiated by two major pathways, intrinsic and extrinsic pathways [8]. The intrinsic pathway, also called the mitochondrial pathway, can trigger cytochrome *c* release from the mitochondria. The extrinsic pathway, also called the death receptor pathway, is activated by death receptors upon ligand binding. Caspase activation is a common characteristic of the two main apoptotic pathways. However, caspase-independent cell death has been reported, which is defined as cell death that does not involve caspase activation [9], [10]. Recently, in addition to the known mitochondrial proteins which is associated with caspase activation, some mitochondrial proteins have been found to activate caspase-independent cell death, including the apoptosis-inducing factor (AIF), endonuclease G (Endo G), and HtrA2 (Omi).

Survivin is a bi-functional member of the inhibitor of the apoptosis protein (IAP) family that mediates cell survival and controls cell cycle progression [11]–[13]. It is not found in normal differentiated tissues but it is usually over-expressed in human cancer tissues, especially in leukemia tissues [14]–[16]. When cells are exposed to stimuli such as Fas/CD95, radiation or chemotherapy, survivin can protect them from cell death. In addition, survivin can inhibit caspase-9 directly and caspase-3 and -7 indirectly by binding to Smac/Diablo [17]. In addition to its role in the caspase-dependent pathway, survivin has been reported to affect the caspase-independent pathway by suppressing AIF release, which provides an alternative route to cell survival [12]. Survivin expression is also up-regulated in the G_2_/M phase of proliferating cells [18]. It is a member of the chromosome passenger complex and plays an important role in cell division. Moreover, many other factors such as Wnt/β-catenin, cytokines, AKT, and NF-κB have been reported to up-regulate survivin expression independent of the cell cycle [12].

The purpose of this study was to investigate the molecular mechanisms underlying the activity of α-tomatine, which was isolated from tomato, and to determine its possible role in leukemia treatment. Our results show that α-tomatine has anti-cancer activity, both *in vitro* and *in vivo*. α-Tomatine exposure induced leukemic cell death independent of cell cycle progression and caspase activation. However, it caused Bak and Mcl-1s activation and subsequently loss of membrane potential, triggering AIF release. It also inhibited the expression of survivin, both *in vitro* and *in vivo*. These findings suggest that α-tomatine may be a promising candidate drug for the treatment of leukemia.

## Materials and Methods

### Materials

α-Tomatine was purchased from Tokyo Chemical Industry Co., Ltd. and dissolved in DMSO (dimethysulfoxide), then kept at −20°C. RPMI 1640, fetal bovine serum (FBS), penicillin/streptomycin were purchased from Life Technologies (Grand Island, NY, USA). Doxorubicin, Z-VAD-FMK, rhodamine 123, 3-(4, 5-dimethylthiazol-2-yl)-2, 5 -diphenyltetrazolium, propidium iodide and all of the other chemical reagents used in this study were purchased from Sigma Chemical (St. Louis, MO, USA). Annexin V-FITC Apoptosis Detection Kit and antibodies against caspase-6 and caspase-7 were purchased from BD Bioscience (San Jos, CS, USA). Antibody against caspase-3 was purchased from Imgenex (San Diego, CA, USA). Antibodies against caspase-8, survivin, AIF, and Bid were purchased from Cell Signaling (Beverly, MA). Antibody against actin was purchased from Millipore (Billerica, MA, USA). Antibody against caspase-9 was purchased from Epitomics (Burlingame, CA, USA). α-Tubulin, poly(ADP-ribose) polymerase (PARP), Mcl-1, Bcl-2, Bak, Bcl-xl, HRP-conjugated anti-mouse and anti-rabbit were purchased from Santa Cruz (CA, USA).

### Cell lines

Human chronic myeloid leukemia K562 cell line and acute promyelocytic leukemia HL60 cell line were obtained from Bioresource Collection and Research Center. Both of them were grown in RPMI-1640 medium with 10% fetal bovine serum, 100 U/mL penicillin, and 100 µg/mL streptomycin and maintained in 5% CO_2_ at 37°C.

### Cell viability assay

Cell viability was determined by MTT assay. The mitochondrial dehydrogenase in living cells reduced 3-(4, 5-dimethylthiazol-2-yl)-2, 5-diphenyltetrazolium bromide, MTT (yellow) to formazan dyes (purple). K562 cells (3×10^5^/mL) and HL60 cells (4×10^5^/mL) were seeded onto 24-well plate. The cells were treated with α-tomatine for 24 hr. After treatment with drug, 100 µL MTT solution (0.5 mg/mL in PBS) per well was added to 24-well plate and the plate was incubated at 37°C for 1 hr. Finally, 100 µL extraction buffer (0.1 M sodium acetate buffer) was added to the plate per well to dissolve the formazan dyes and the absorbance was measured at 550 nm by ELISA reader (Packard, Meriden, CT, USA).

### Flow cytometry analysis

The phenomenon of apoptosis was detected by phosphatidylserine (PS) translocating from the inner membrane to the outer cell surface. And labeled annexin V can bind to PS to detect apoptotic cells. After cells were treated with α-tomatine during the time, cells were harvested and stained by propidium iodide (PI, 0.5 µg/mL) and Annexin V-FITC (25 µg/mL) solution for 15 min at room temperature. The percentage of apoptotic cells were analyzed by FACScan Flow Cytometer and CellQuest software (Bectan Dickinson). DNA content can be used to analyze the cell cycle. Following treatment with α-tomatine during the indicated time, cells (5×10^5^/mL) were harvested and fixed with 70% (v/v) ice cold ethanol at −20°C for 30 min at least. The fixed cells were rinsed twice with phosphate-buffered saline (PBS), resuspensed in 0.2 mL DNA extraction buffer (0.2 M Na_2_HPO_4_-0.1 M citritic buffer, pH 7.8) for 30 min and stained with PI solution (80 µg/mL propidium iodide, 100 µg/mL RNase A, and 1% Triton X-100 in PBS) for 30 min at room temperature. Data were analyzed by FACScan Flow Cytometer and CellQuest software (Bectman Dickinson).

### Measurement of mitochondrial membrane potential

The mitochondrial membrane potential was monitored by rhodamine 123. Rhodamine 123, a kind of lipophilic fluorone dye with negative charged, can be selectively absorbed into mitochondrial membrane. When mitochondrial membrane potential is lost, rhodamine 123 can't be absorbed into membrane. Cells were treated with the α-tomatine for the indicated time. Rhodamine 123 (final concentration 10 µM) was added before the cells were harvested and incubated for 30 min at 37°C. Then the cells were finally collected, rinsed with PBS and analyzed by FACScan Flow Cytometer and CellQuest software (Bectman Dickinson).

### Western blot analysis

After the treatment, cells (10^6^cells/mL) were harvested. Whole cell pellets were washed twice with PBS, lysed in ice-cold lysis buffer (50 mM Tris, pH 7.4, 150 mM NaCl, 1% Triton X-100, 1 mM EDTA, 1 mM EGTA, 1 mM PMSF, 10 µg/ml aprotinin, 10 µg/ml leupeptin, 1 mM sodium orthovandate and 1 mM NaF) for 30 min and subsequently centrifuged at 13,000 rpm at 4°C for 30 min. For extraction of nuclear protein, cell pellets were lysed in buffer A (10 mM Hepes, pH 7.9, 10 mM KCl, 1.5 mM MgCl_2_, 0.2 mM PMSF, 0.5 mM DTT, dH_2_O). After incubation on ice for 15 min, cells were centrifuged at 2,000 rpm at 4°C for 3 min and then the pellets were rinsed with buffer B (10 mM Hepes, pH 7.9, 50 mM NaCl, 0.1 mM EDTA, 25% glycerol, dH_2_O). Finally, the pellets were resuspended in buffer C(20 mM Hepes, pH 7.9, 420 mM NaCl, 1.5 mM MgCl_2_, 0.2 mM EDTA, 25% glycerol, 0.2 mM PMSF, 0.5 mM DTT) for 20 min on ice and centrifuged at 13,000 rpm at 4°C for 30 min. Protein was quantified by BCA Protein Assay Kit (Thermo scientific, Rockford, IL, USA). For Western blot analysis, the protein was separated by electrophoresis and transferred onto a nitrocellulose membrane. Then blocking the membrane with nonfat milk for 1 hr and was incubated with primary antibody in PBS at 4°C overnight. On the next day, the membrane was washed with PBST (0.1% Tween 20 in PBS) and incubated with secondary antibody for 1 hr at room temperature. Then the membrane was washed again with PBST and finally the signal was detected with an enhanced chemiluminescence detection kit (Amersham, Buckinghamshire, UK).

### Tumor xenograft models

To estimate the *in vivo* antitumor activity of α-tomatine, HL-60 cells (10^8^ cells/mL) were injected into 20 severe combined immunodeficient (SCID) mice subcutaneously. When average tumor size approximately reached 100 mm^3^, mice were separated to two groups (one group for 10 mice) and then treated with α-tomatine (5 mg/kg) in 5% DMSO/5% Cremophor/90% D5W (5% glucose) intraperitoneally. Once average size of the tumor was greater than 2,500 mm^3^, mice were sacrificed. Tumors were resected, weighed, and frozen in formalin for immunohistochemical experiments. Tumor size was measured by caliper measurement (mm) and ellipsoid sphere formula (LW^2^/2, L: length; W: width). All procedures were followed by National Taiwan University Animal Use and Management Committee.

### Immunohistochemical Staining

Paraffin-embedded tumor tissues from mice were sectioned and deparaffinized with xylene. The slides were immersed into different concentration of alcohol (100%, 95%, 75%, 50%, ddH_2_0) step by step for rehydration and then in 3% H_2_0_2_ to block endogenous peroxidase. For antigen retrieval, immersing the slides in boiling (95–100°C) citrate buffer (pH 6.0) for 20 min was required. After washing with PBS, the slides were soaked in blocking solution (3% BSA) at room temperature for 30 min. The slides were incubated with the diluted primary antibody, survivin or AIF (Cell Signaling, Beverly, MA) at 4°C overnight. After rinsing with PBS for several times, the secondary antibody, HRP Polymer Conjugate Reagent (SuperPicture Polymer Detection kit), was added for 10 min and then DAB Chromogen for 5 min. Each incubation step, the slides were followed by washing with PBS for 5 min. And then, Mayer's Hematoxylin solution was used for counterstaining. Finally, the slides had to be dehydrated, air-dried and mounted. For hematoxylin and eosin staining (H&E stain), in briefly, the sectioned slides were placed in hematoxylin solution for 15 min, washed with ddH_2_O and then counterstained with eosin for 5 min. The color of nuclei of cells was blue and of cytoplasm was pink. This kind of staining can distinguish the nuclei and cytoplasm of tumor cells.

### Statistical analysis

All experimental data were expressed as mean values ± SEM and assessed by Bonferroni *t*-test. The animal experiments were done by the Mann-Whitney test. Statistical significance was determined by *P*<0.05.

## Results

### α-Tomatine induced apoptosis in HL60 and K562 leukemia cell lines

**Figure 1:**
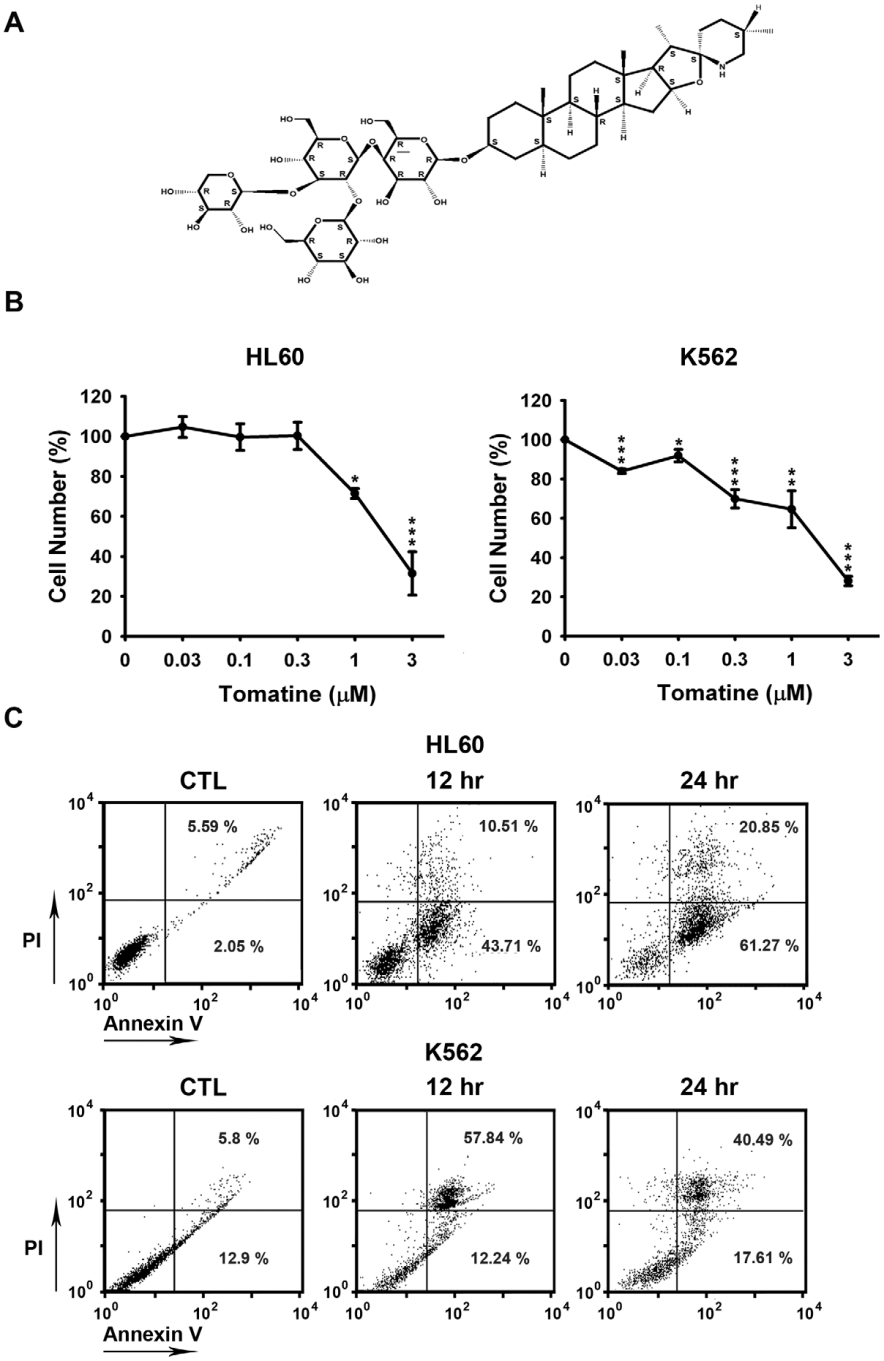
α-Tomatine-induced apoptosis in human leukemia cell lines. (**A**) The chemical structure of α-tomatine. (**B**) Cells were treated with or without α-tomatine for 24 hr, and cell viability was measured by using the mitochrondrial MTT reduction activity assay. Data are expressed as the means ± SEM of at least three determinations. * *P*<0.05, ** *P*<0.01, and *** *P*<0.001 compared with the control. (**C**) Flow cytometry analysis of plasma membranes with Annexin V-FITC/PI double staining. Cells were incubated with DMSO for 12 hr or in the presence of 5 µM α-tomatine for 12 and 24 hr. In the following experiments, 0.1% DMSO was used as control. Undamaged cells were stained negative by Annexin V-FITC/PI (bottom left quadrant). After incubation with 5 µM of α-tomatine for 12 hr, there were a significant number of apoptotic cells that stained positive with Annexin V-FITC and negative with PI (bottom right quadrant). Data are expressed from at least three separate determinations.

α-Tomatine is composed of steroid-like tomatidine, galactose, glucose, and xylose (Fig. 1A). The cytotoxicity of α-tomatine was first determined with two different types of human leukemic cells, K562 (human chronic myeloid leukemia) and HL60 (human acute promyelocytic leukemia). MTT assay revealed α-tomatine had strong cytotoxic effects that could inhibit cell survival in HL60 and K562 in a concentration-dependent manner at IC_50_ of 1.92 and 1.51 µM, respectively (Fig. 1B). These results were also shown in other leukemia cell lines (Fig. S1). However, α-tomatine had rather weak cytotoxic effect on lymphoma cell line and normal cells (Fig. S1 and S2). To confirm the status of the cell death process induced by α-tomatine, PI and Annexin V double staining were performed. As shown in Figure 1C, approximately 40% of HL60 cells treated with α-tomatine for 12 hr were stained by Annexin V, which represented early stage apoptotic cells, and 10% of the cells were stained by both PI and Annexin V, which indicated late phase apoptosis or necrosis. After treatment for 24 hr, 60% of the cells were in the early phase of apoptosis and 20% of the cells were in the late phase. These findings indicate that α-tomatine could inhibit cell growth and induce apoptosis in the HL60 and K562 cell lines.

### α-Tomatine did not affect the cell cycle distribution

**Figure 2:**
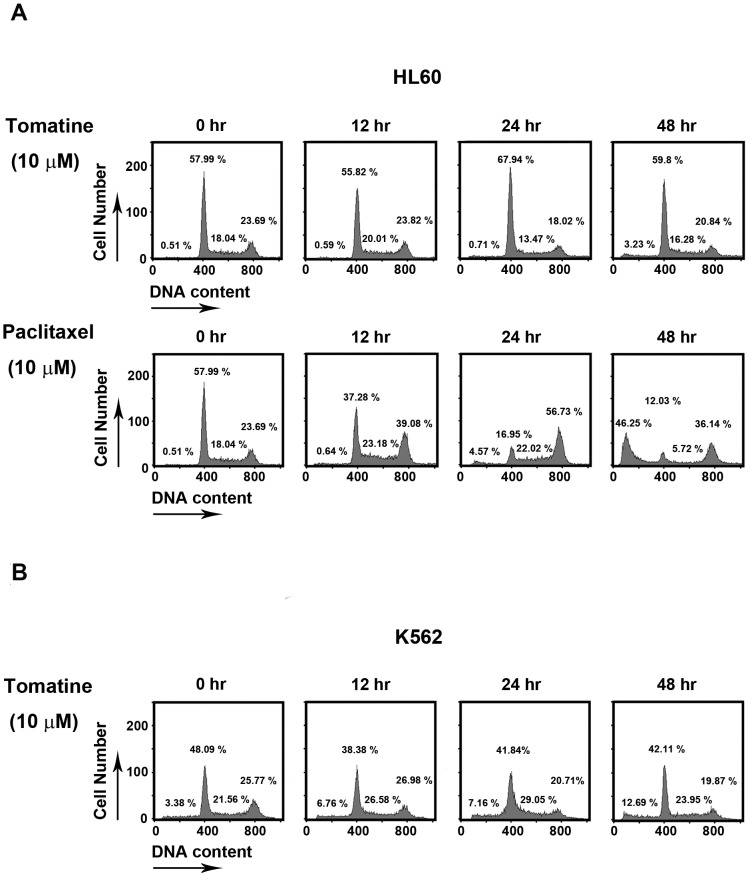
Cell cycle distribution of α-tomatine in HL60 and K562 cell lines. (**A**) The upper lane shows HL60 cells that were treated with α-tomatine (10 µM) for the indicated time; the cell cycle distribution was assessed by FACScan flow cytometric analysis. The bottom lane, HL60 cells treated with paclitaxel (10 µM) for the indicated time, served as a positive control. (**B**) K562 cells were treated with α-tomatine (10 µM) for the indicated time. Data are expressed from at least three separate determinations.

Analysis of the cell cycle phase distribution could help evaluate α-tomatine's mechanism of action; to observe cell cycle distribution for both HL60 and K562 cell lines, α-tomatine was used at 10 µM for 12, 24, and 48 hr. No obvious cell cycle phase changes were observed with regard to time-dependent exposure to α-tomatine (Fig. 2A and 2B). These findings suggest that the phase of the cell cycle for the HL60 and K562 cell lines was not altered after α-tomatine treatment. In other leukemia cell lines, α-tomatine also did not have influence on cell cycle distribution (Fig. S3). According to previous studies, cell cycle accumulation in the G_2_/M phase was observed with paclitaxel [19]. Paclitaxel was used as a positive control. Flow cytometry showed that the proportion of cells in the G_0_/G_1_ phase decreased and sub-G_1_ population increased after treatment of the cells with paclitaxel at a dose of 10 µM. After treatment for 24 hr, compared to the control group cells, the number of cells in the sub-G_1_ phase increased approximately 15-fold (Fig. 2A). These findings suggest that α-tomatine did not affect the cell cycle distribution of the human leukemia cells studied.

### α-Tomatine induced cell death independent of caspase activation

**Figure 3:**
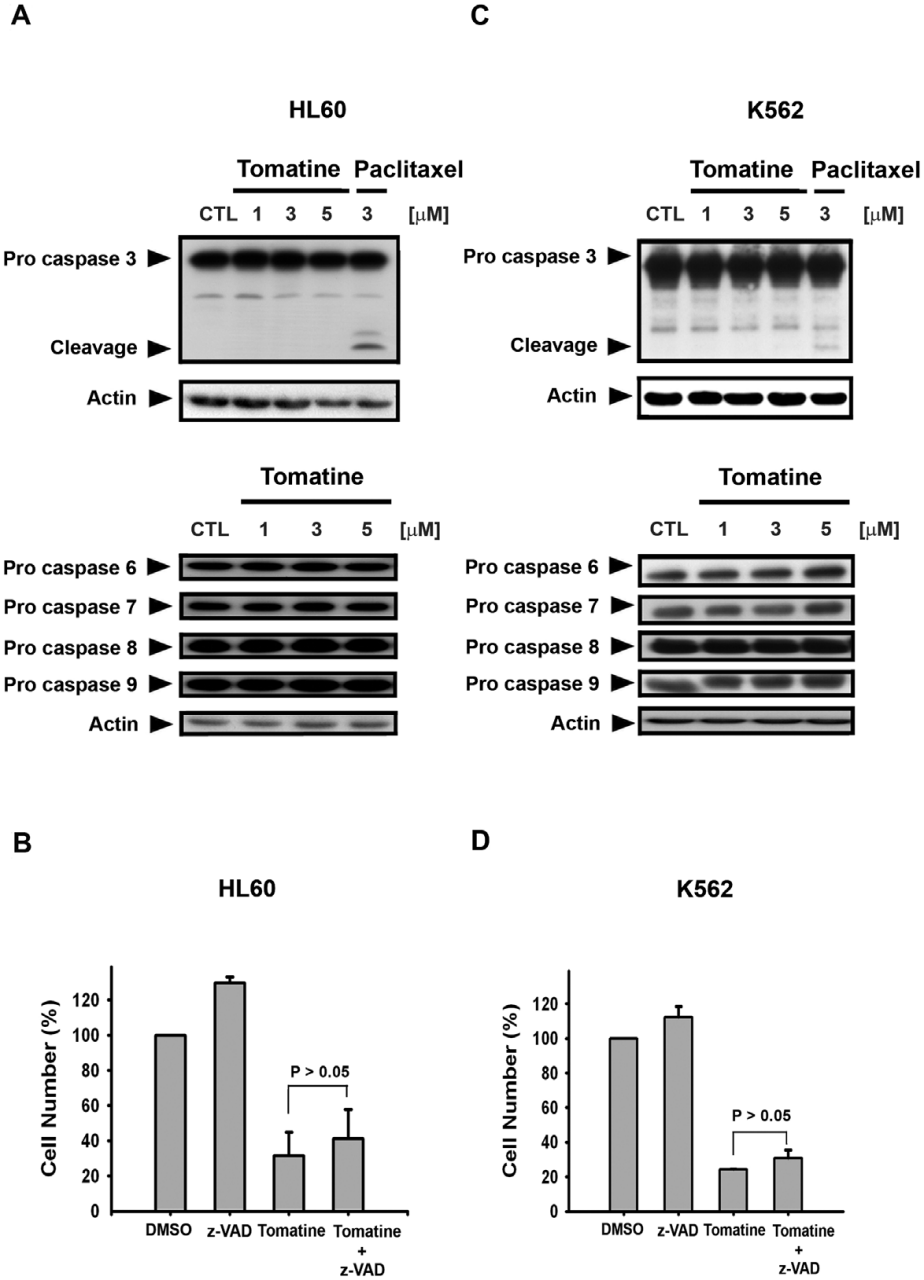
α-Tomatine induced cell death independent of caspase activation in both K562 and HL-60 cell lines. (**A**) HL60 cells were treated with α-tomatine (5 µM) or paclitaxel (3 µM) for 24 hr and caspase-3, -6, -7, -8, and -9 activations were detected. The proteins were separated and evaluated using Western blot analysis. Paclitaxel (3 µM) was used as a positive control. (**B**) HL60 cells were pretreated with 100 µM z-VAD-fmk for 30 min and then treated with α-tomatine (5 µM) for 24 hr. The cytotoxicity was determined by MTT assay. (**C**) K562 cells were treated with α-tomatine (5 µM) and caspase-3, -6, -7, -8, and -9 activations were detected. (**D**) K562 cells were pretreated with 100 µM z-VAD-fmk for 30 min and then treated with α-tomatine (5 µM) for 24 hr.

Caspase activation plays an important role in both intrinsic and extrinsic apoptotic pathways. Among all caspases, activation of caspases-3, 6, and-7 is thought to be the most important in the apoptosis pathway. Therefore, to confirm the role of caspase-3 the HL60 and K562 cells were treated with α-tomatine at different concentrations. The results showed that caspase-3 is not activated by α-tomatine (Fig. 3A and 3C). However, paclitaxel (3 µM), used as a positive control, activated caspase-3. In addition to caspase-3, caspase-6, -7, -8, and -9 were apparently unchanged after α-tomatine treatment, even after exposure to a high concentration of α-tomatine (5 µM) for 24 hr (Fig. 3A and 3C). In order to confirm that all of the caspase activation was involved in α-tomatine-induced cell death pathways, a pan caspase inhibitor, z-VAD-fmk, was used for the confirmation experiments. MTT assay showed that co-treatment of HL60 and K562 cells with z-VAD-fmk and α-tomatine did not reverse α-tomatine-induced cell death (Fig. 3B and 3D). In addition, these similar findings were also displayed in other leukemia cell lines (Fig. S4). These results suggest that α-tomatine induced cell apoptosis is independent of caspase activation in the leukemia cell lines.

### α-Tomatine affected the mitochondrial membrane potential and related proteins

**Figure 4:**
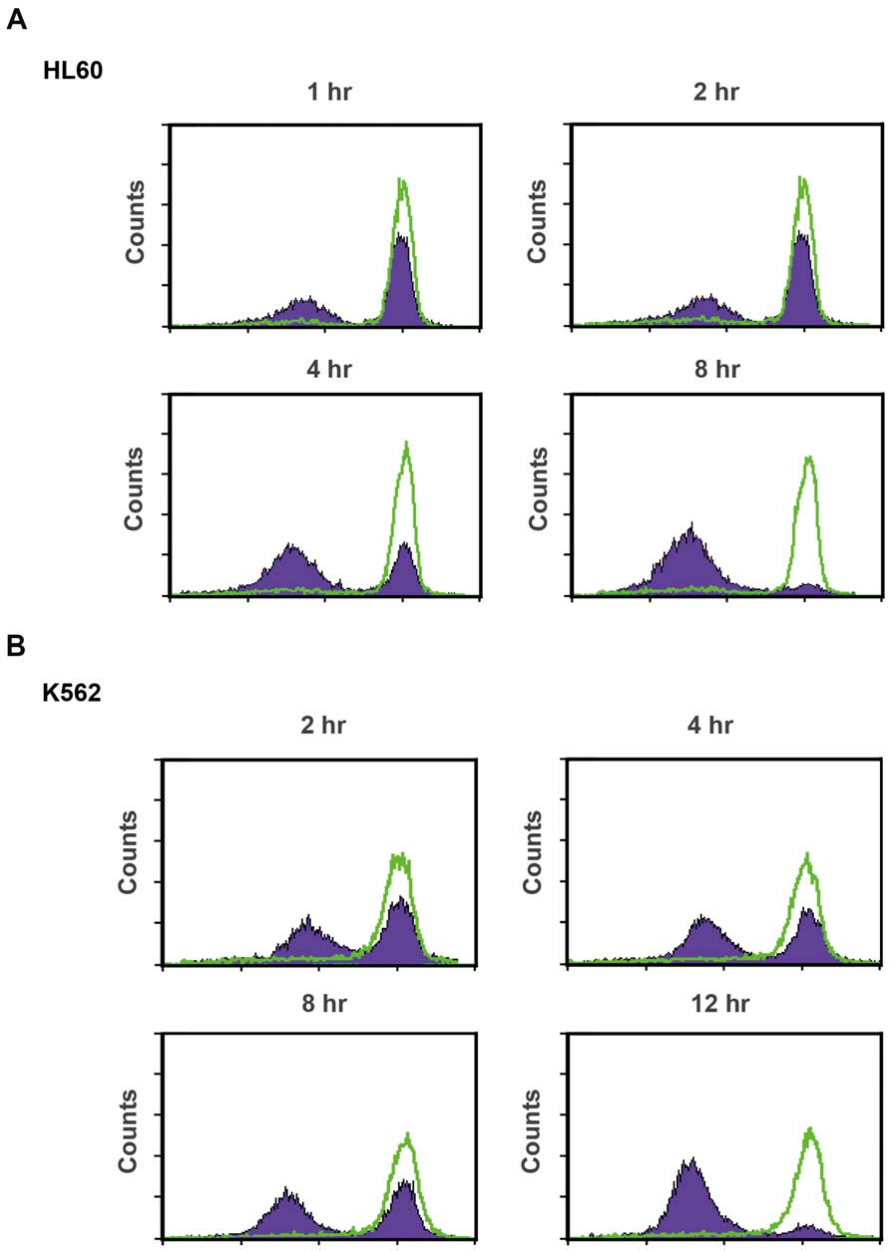
Effects of α-tomatine on the mitochondrial membrane potential in both HL60 and K562 cell lines. The mitochondrial membrane potential was quantitated by flow cytometric analysis with rhodamine 123. The (**A**) HL60 and (**B**) K562 cell lines were treated with 10 µM rhodamine 123 and incubated at 37°C for 30 min in the presence of 5 µM α-tomatine. The horizontal axis shows the relative fluorescence intensity, and the vertical axis indicates the cell number. The green curve indicates the control. The blue curve indicates the α-tomatine-treated cells. A shift from the green curve to the blue curve indicates a loss of mitochondrial membrane potential. Data are expressed from at least three separate determinations.

Because mitochondria play an important role in both intrinsic and extrinsic apoptosis pathways, mitochondrial membrane potential was measured. HL60 and K562 cells were exposed to α-tomatine at a concentration of 5 µM for the indicated times (1, 2, 4, 8, and 12 hr) and treated with rhodamine 123 for 30 min; and then, the cells were analyzed by flow cytometry. Figures 4A and 4B show a band shift phenomenon observed after incubation for 1 and 2 hr in the HL60 and K562 cell lines, respectively; these phenomena changed significantly at 4 hr. The above mentioned indicate that α-tomatine affected the mitochondrial membrane potential. Not only in the HL60 and K562 cells, α-tomatine also caused mitochondrial membrane potential loss in other leukemic cells (Fig. S5).

**Figure 5:**
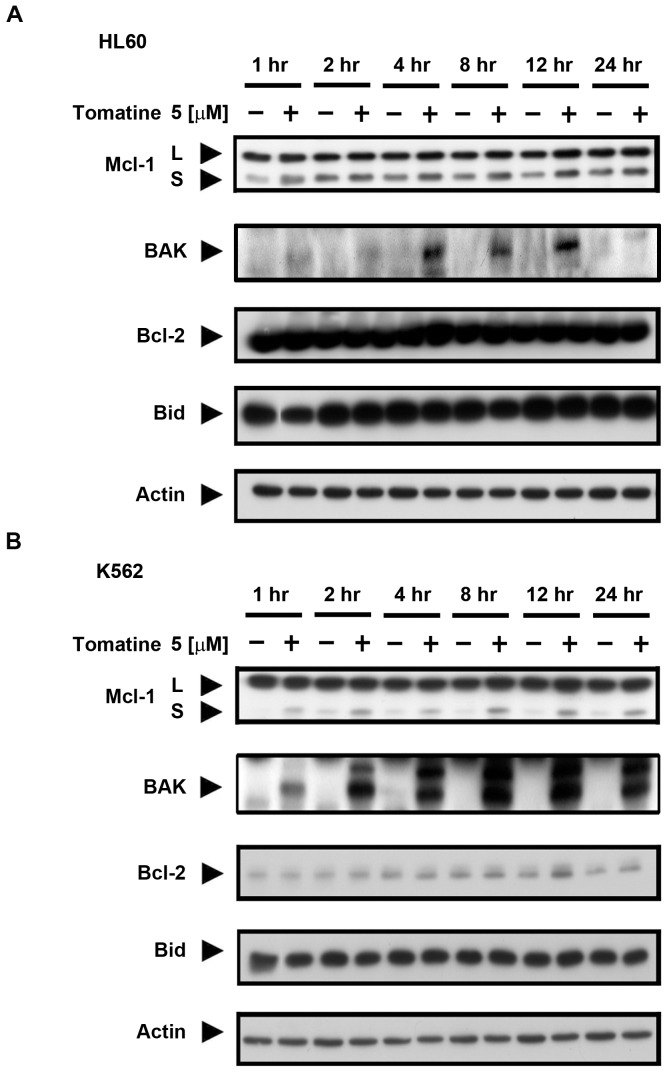
α-Tomatine affected mitochondrial apoptotic or anti-apoptotic protein levels. (**A**) α-Tomatine induced Mcl-1s and Bak up-regulations (pro-apoptotic) but did not affect Bcl-2 and Bid protein levels in the HL60 cells. (**B**) In the K562 cells, α-tomatine significantly enhanced the activation of Bak and up-regulated Mcl-1s; however α-tomatine did not influence Bcl-2 and Bid protein expressions. Both cell lines were treated with 5 µM α-tomatine for the indicated intervals. Data are expressed from at least three separate determinations.

In previous studies, the Bcl-2 family was shown to play a crucial role in the mitochondria, including mitochondrial membrane potential mediation [20]. Therefore, we investigated whether α-tomatine induced changes in the mitochondrial membrane potential because of the effects of the Bcl-2 family protein expression. In the HL60 and K562 cells, the Mcl-1 long form (Mcl-1L) protein, which mediates inhibition of cell apoptosis, was not regulated by α-tomatine; however, expression of the Mcl-1 short form (Mcl-1s), which induces cell apoptosis, increased following treatment (Fig. 5A and 5B). Nevertheless, as shown in Figures 5A and 5B, there were no changes in the Bcl-2 and Bid protein levels in the HL60 and K562 cells. These findings suggest Bak and Mcl-1 played important roles in α-tomatine-induced apoptosis in human leukemia cells.

### α-Tomatine induced AIF nuclear translocation and inhibited survivin expression

**Figure 6:**
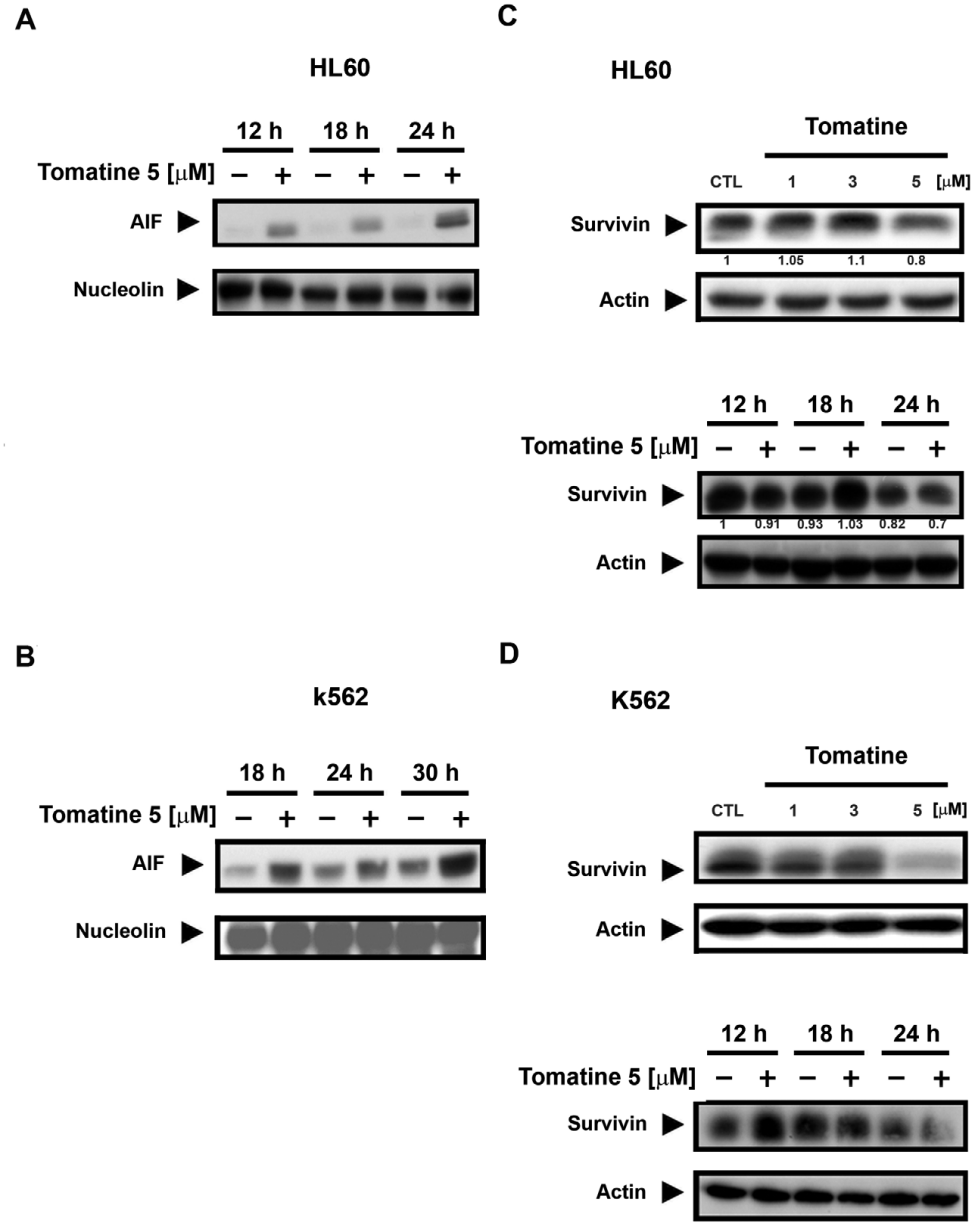
α-Tomatine induced nuclear translocation of AIF and survivin down-regulation in both HL60 and K562 cell lines. (**A**) HL60 and (**B**) K562 cells were treated with and without α-tomatine (5 µM) for 12 hr, 18 hr, 24 hr, and 30 hr. Cells were then fractionated into nuclear components, and the protein expressions of AIF and nucleolin (nuclear loading control) were evaluated by Western blot analysis. (**C**) HL60 and (**D**) K562 cells were treated with α-tomatine at the indicated concentrations and time. Survivin and actin protein levels were detected by Western blot analysis. Data are expressed from at least three separate determinations.

Previous findings and the data presented here have revealed that α-tomatine induced cell death independent of caspase activation [21] and affected mitochondrial membrane potential. Moreover, it has been reported that the permeability of the mitochondrial membrane is also regulated by AIF. Thus, AIF-induced cell death, which bypasses caspase activation, may be involved in the α-tomatine-induced cell apoptosis pathway. Figure 6A and 6B show α-tomatine-induced nuclear translocation of AIF in both cell lines in a time-dependent manner.

According to Carter *et al.*, many types of leukemia cell lines are associated with survivin inhibition that promotes cell death independently of cell cycle progression [22]. In addition, survivin has been shown to suppress AIF translocation to the cytoplasm from the mitochondria that could induce the caspase-independent apoptotic pathway [12]. We investigated whether α-tomatine could directly down-regulate the survivin protein level that leads to cell death. Indeed, the expression of survivin was down-regulated in both cell lines and other leukemia cell lines in a concentration- and time-dependent manner (Fig. 6C and 6D and S6). These findings indicate that AIF translocation and survivin inhibition were involved in α-tomatine-mediated caspase-independent cell death.

### Antitumor activity and expression of AIF and survivin with α-tomatine *in vivo*

**Figure 7:**
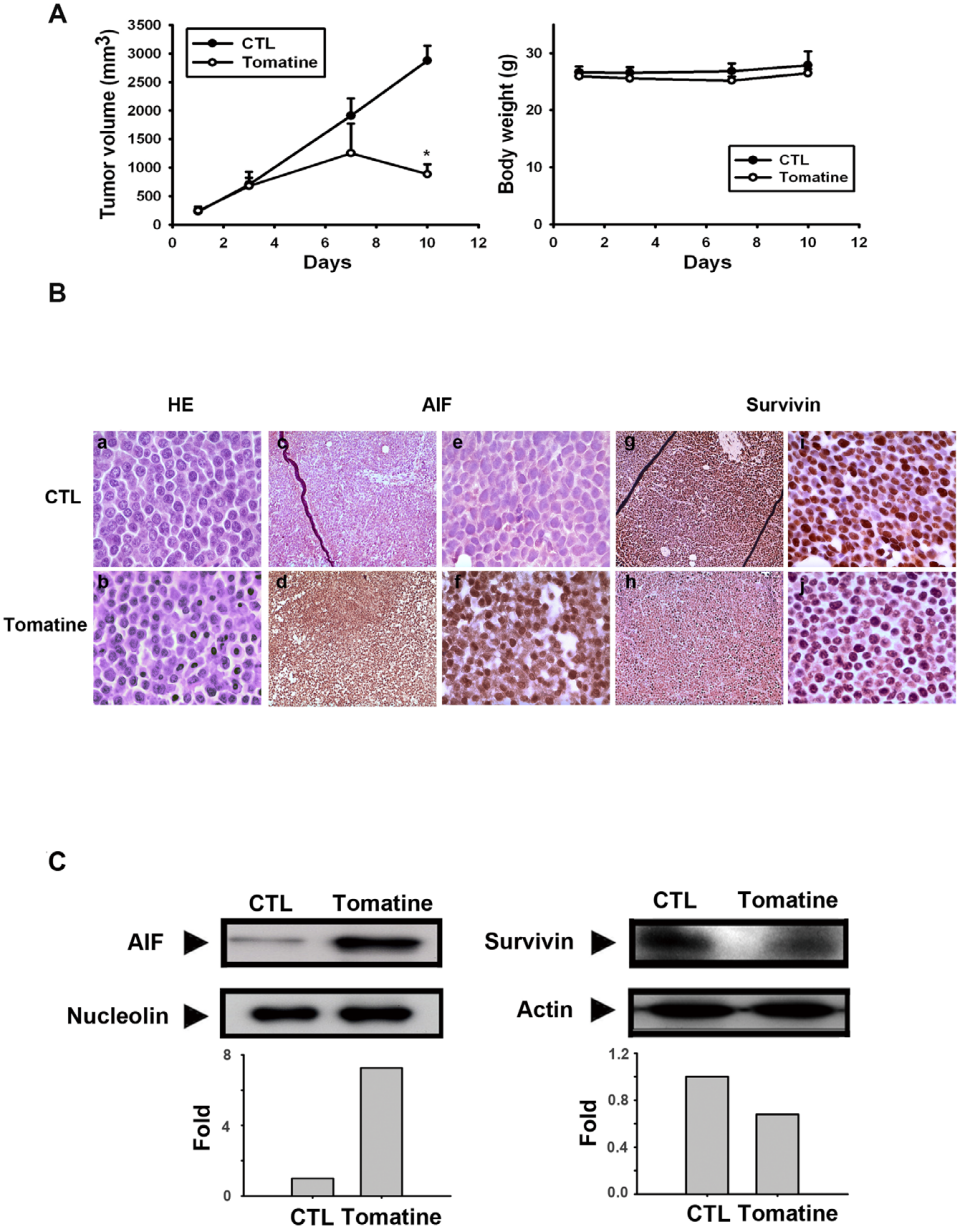
α-Tomatine significantly inhibited HL60 xenograft tumor growth and affected AIF and survivin expression *in vivo*. HL-60 cells were ectopically implanted into SCID mice and when the tumor size reached 100 mm^3^, the mice were injected with 5 mg/kg (q2d, i.p.) doses of α-tomatine. (**A**) Effects of α-tomatine on tumor volume and the body weights of mice were studied. The growth curves are the means of the tumor sizes measured for each group (n = 5). (**B**) The tumors were then excised and processed for immunohistochemical staining. The upper lanes (a.c.e.g and i) are the control, and the down lanes (b.d.f.h and j) are the treated group, with α-tomatine (5 mg/kg). a,b: Hematoxylin and eosin staining; c,d,e and f staining for AIF (brown) ; g,h,i and j staining for survivin (brown). c,d,g and h are under 200× magnification; a,b,e,f and j are under 1000× magnification. (**C**) Western blot analysis was performed for AIF and survivin expressions together with actin as a loading control from randomly selected tumor in each of the control and 5 mg/kg α-tomatine treatment groups.

To determine the antitumor efficacy of α-tomatine in *vivo*, SCID mice were subcutaneously injected with HL60 cells in their right flank. When the tumor volume reached 100 mm^3^, the mice were divided into two groups: a control (vehicle) and α-tomatine treatment groups (5 mg/kg, i.p., every other day). The experiment was discontinued when the average volume of tumors reached 2,500 mm^3^. Figure 7A shows that α-tomatine inhibited tumor growth and was associated with lack of weight loss in the treated mice. The tumors were resected; one part of the resected tumors was fixed in formalin and embedded in paraffin for immunohistochemical analysis, and the other part of was ground and then immersed into lysis buffer. Immunohistochemical staining was performed to estimate the expressions of AIF and survivin, *ex vivo*. Hematoxylin and eosin (H&E) staining was used to observe the appearance of the cells, with the blue and red portions representing the cell nucleus and cytoplasm, respectively. The nuclei shrank in the tumor cells of the α-tomatine-treated mice (Fig. 7B). Results of immunohistochemical staining used to assess the AIF and survivin expression levels showed that, compared to the vehicle-treated mice, α-tomatine-treated mice showed increased AIF expression and reduced survivin levels (Fig. 7B). In addition, Western blot analysis was used to measure a portion of the ground tumor tissue for the expressions of AIF and survivin. Figure 7C shows that, consistent with the observed effects of α-tomatine *ex vivo*, the AIF protein level was increased and survivin was decreased in the α-tomatine-treated mice. Taken together, these findings indicate that α-tomatine, both *in vitro* and *in vivo*, inhibited leukemia cell growth by survivin inhibition and AIF induction suggesting that α-tomatine may be a candidate for effective leukemia treatment.

## Discussion

α-Tomatine is a major saponin found in the tomato, and recent studies have shown that it has antitumor activity in solid tumor cells [1], [5], [6]. However, the molecular mechanism of α-tomatine activity has not been determined in leukemic cells. In the present study, α-tomatine was found to inhibit the survival of leukemic cells in a concentration-dependent manner (Fig. 1B and S1). The inhibition of cell survival might be attributed to cell growth inhibition or cell cytotoxicity. Therefore, the cell cycle distribution was investigated further after treatment with α-tomatine. The results showed that α-tomatine did not affect cell cycle progression in the evaluated leukemia cell lines (Fig. 2 and S3). However, propidium iodide (PI) and Annexin V staining revealed that α-tomatine promoted-leukemic cell apoptosis from the early to the late phases (Fig. 1C). Moreover, α-tomatine treatment resulted in changes in the expression of the BCL-2 protein family (Fig. 5). Significant mitochondrial perturbation (Fig. 4 and S5) was observed, and it subsequently triggered AIF translocation to the nucleus and inhibited survivin expression leading to leukemic cell apoptosis (Fig. 6 and S6).

The results of this study showed no α-tomatine-related changes in the distribution of the cell cycle, even at a high α-tomatine concentration (10 µM). However, the results of PI-annexin V double staining suggested that the apoptosis observed was caused by α-tomatine. Further, Annexin V staining showed that the cell membrane was still intact in the early phase of apoptosis, but phosphatidylserine, which is normally observed on the inner leaflet of the membrane, was translocated to the outer membrane. In the late phase of apoptosis, the cell membrane was disrupted and PI leaked into the cells and bound to the DNA. The percent of cell apoptosis after treatment of the cells with α-tomatine was estimated by calculating the amount of Annexin V stain in the cells. Moreover, the DNA levels during the cell cycle were determined on the basis of PI staining, since PI bound to the DNA in the nucleus. The fluorescence observed increased with the increase in the percent of PI and DNA binding. However, further study is needed to explain the specifics of α-tomatine-induced cell death observed on PI-Annexin V double staining in cells without any change in the cell cycle phases.

Caspase activation plays a critical role in the classical apoptosis pathway. The initiator caspases, such as caspase-8 and -9, play important roles in both the extrinsic and intrinsic apoptotic pathways. Caspase-3, -6, and -7 are the downstream effector caspases that cleave poly (ADP-ribose) polymerase (PARP), resulting in cell apoptosis. After α-tomatine treatment, the caspases were not activated; the α-tomatine-induced cell death was not reversed even when the cells were pretreated with the pan-caspase inhibitor z-VAD-fmk. These findings support the observation that the α-tomatine-induced cell death was independent of caspase activation. A prior study showed that α-tomatine-induced EL4 thymoma cell death was independent of caspase activation [4]; this may be a unique characteristic of α-tomatine. Furthermore, many previous studies have shown that many natural products do not have to activate caspases to induce leukemic cell death. For example, Reo *et al.* reported that catechin extracted from green tea was associated with caspase-independent cell death in chronic myeloid leukemia cells [23]; Lipoic acid, a potential antioxidant, often used in foods, also induced caspase-independent cell death in the acute promyeloid leukemia cell line HL60 [24].

The effects of α-tomatine on the mitochondria were also studied to determine whether induction of AIF release and apoptosis proceeded independently of caspase activation. The results showed that treatment with α-tomatine was associated with mitochondrial membrane potential loss and the concomitant induction of the expression of Bak, a member of the pro-apoptotic Bcl-2 family, and Mcl-1s, an anti-apoptotic Mcl-1 short form. Donovan *et al.* reported that the release of mitochondrial proteins could be explained by the following two types of models [25]. The first type of model is associated with the pro-apoptotic Bax and Bak-forming channels that trigger the release of the protein in the mitochondria to the cytoplasm and then activate caspase-dependent or caspase-independent cell apoptosis. Mitochondrial proteins, especially large-molecular-weight proteins, were associated with the caspase-independent pathway, including AIF (57 kDa), endoG (30 kDa), and Omi/HtrA2 (30 kDa) that may pass through larger channels formed by Bax and voltage-dependent anion channel (VDAC). The second model is associated with the permeability transition pore (PTP) complex opening, with mitochondrial membrane potential loss and changes in the permeability, resulting in mitochondrial release of the protein. The PTP is regulated by the pro-apoptotic Bcl-2 family, Bax and Bak or by the anti-apoptotic Bcl-2 and Bcl-xl. Therefore, α-tomatine might have an effect on Bak and Mcl-1 or directly activate the PTP complex to change the mitochondrial membrane potential.

Mcl-1 is an anti-apoptotic protein that belongs to the Bcl-2 family. In general, the Mcl-1 long form (Mcl-1L) function has been associated with anti-apoptotic activity. When Mcl-1L is inhibited, apoptosis of cells occurs. Recently the short form of Mcl-1 (Mcl-s) has been shown to have the opposite effect, induction of apoptosis in cells [26]. Huang *et al.* reported that caseamembrin C extracted from *Casearia membranase*) was associated with expression of the protein Mcl-1s [27]. In addition, Bak activity has been reported to be regulated by the anti-apoptotic Mcl-1 and Bcl-xl [28]. The results of this study showed that pro-apoptotic Mcl-1s was induced and Bak was activated after treatment with α-tomatine.

Many studies have suggested that AIF is a very important mitochondrial protein in caspase-independent cell death [29]. Release of AIF from the mitochondria is followed by direct entry into the cell nucleus, resulting in DNA fragmentation, which in turn leads to cell apoptosis. The observation of that α-tomatine-induced cell death that was independent of caspase activation, led to the evaluation of AIF in this pathway. The results showed that the AIF protein level of the nucleus increased with α-tomatine, thus promoting leukemic cell apoptosis.

In a recent study, it was reported that survivin is an important mediator in the cell cycle-independent apoptosis pathway [30]. Anti-apoptotic survivin plays a crucial role in leukemia. Survivin is usually over expressed in many types of human leukemia cell lines [15]. However, in normal peripheral monocytes and CD34^+^ precursor cells, the protein level of survivin is very low and often undetectable [22]. Survivin can be activated in a cell cycle-dependent or independent manner [30]; therefore, survivin expression with α-tomatine was studied in leukemia cells. The findings showed that α-tomatine significantly suppressed the expression of survivin in leukemia cell lines.

In addition to causing caspase inactivation, survivin inhibits AIF release from the mitochondria into the cytoplasm, in a caspase-independent manner, to induce cell apoptosis [31]. This suggests that both AIF and survivin can regulate the caspase-dependent and caspase-independent pathways. Our results suggest that the α-tomatine-associated decrease in the expression of survivin and increase in the nuclear AIF might be involved in the two different pathways. One pathway is associated with AIF released from the mitochondria directly to the nucleus, resulting in DNA fragmentation. The other pathway may be associated with the inhibition of survivin, increasing AIF translocation to the nucleus. Survivin played a supportive role in this study. However, further investigation is required to better understand the interaction between AIF and survivin.

The results of this study demonstrated that α-tomatine-induced cell death was caspase-independent and associated with survivin inhibition and AIF translocation to the nucleus. An HL60 xenograft animal model was used to confirm that α-tomatine reduces the tumor volume and loss of body weight. Finally, *ex vivo* analysis with immunohistochemistry and immunoblot analysis of AIF and survivin expressions in the tumors excised from the mice studied showed a significant increase in the apoptosis of the α-tomatine-treated group relative to that in the control group. Taken together, the findings of this study support the theory that α-tomatine can play an active role in leukemia cell lines; it inhibited tumor growth in the animal model as well as the *in vitro* experiments. Therefore, α-tomatine may be a novel candidate for the leukemia treatment.

## Supporting Information

Figure S1 **Cell viability of four leukemia and lymphoma cell lines exposed to α-tomatine.** (A) MOLM-13 (acute monocytic leukemia) (B) MV4-11 (acute myelocytic leukemia) (C) THP-1 (acute monocytic leukemia) (D) CCRF-CEM (acute lymphepithelium) (E) BJAB (human lymphoma) cells were treated with or without treated the indicated concentrations of α-tomatine for 24 hr, and cell viability was measured by MTT assay. Data are expressed as the means ± SEM of at least three determinations.
(TIF)

Figure S2 **Cell viability of normal cell lines exposed to α-tomatine.** (A) HUVEC (human umbilical vein endothelial), (B) BEAS (normal human bronchial epithelium), and (C) U937 (normal human monocyte) cells were treated with or without the indicated concentrations of α-tomatine for 24 hr, and cell viability was measured by MTT assay. Data are expressed as the means ± SEM of at least three determinations.
(TIF)

Figure S3 **Cell cycle distribution of α-tomatine in leukemia cell lines.** The indicated cell lines were treated with or without α-tomatine (1, 3, and 10 µM) for 24 hr, and the cell cycle distribution was determined by FACScan flow cytometric analysis. Data are expressed at least three determinations.
(TIF)

Figure S4 **α-Tomatine induced caspase-independent cell death in leukemia cell lines.** The indicated cells were pretreated with 100 µM z-VAD-fmk for 30 min and then treated with or without α-tomatine (5 µM) for 24 hr. The cytotoxicity was determined by MTT assay.
(TIF)

Figure S5 **α-Tomatine affected mitochondrial membrane potential in leukemia cell lines.** The mitochondrial membrane potential was determined by flow cytometric analysis with rhodamine 123. The cells were pre-treated with 10 µM rhodamine 123 for 30 min in the presence of 5 µM α-tomatine. The horizontal axis shows the relative fluorescence intensity, and the vertical axis indicates the cell number. The purple curve indicates the control. The green curve indicates the α-tomatine-treated cells. A shift from the purple curves to the green curves implies a loss of mitochondrial membrane potential. Data are expressed from at least three separate determinations.
(TIF)

Figure S6 **α-Tomatine induced survivin down-regulation in leukemia cell lines.** Cells were treated with or without α-tomatine at the indicated concentrations for 24 hr, and then protein levels were detected by Western blot analysis. Data are expressed from at least three separate determinations.
(TIF)
