# Supplementary figures and images for: α-Tomatine-Mediated Anti-Cancer Activity In Vitro and In Vivo through Cell Cycle- and Caspase-Independent Pathways
Source: PLoS One. 2012 Sep 6;7(9):e44093. doi: 10.1371/journal.pone.0044093 (PMC3435411; doi:10.1371/journal.pone.0044093)

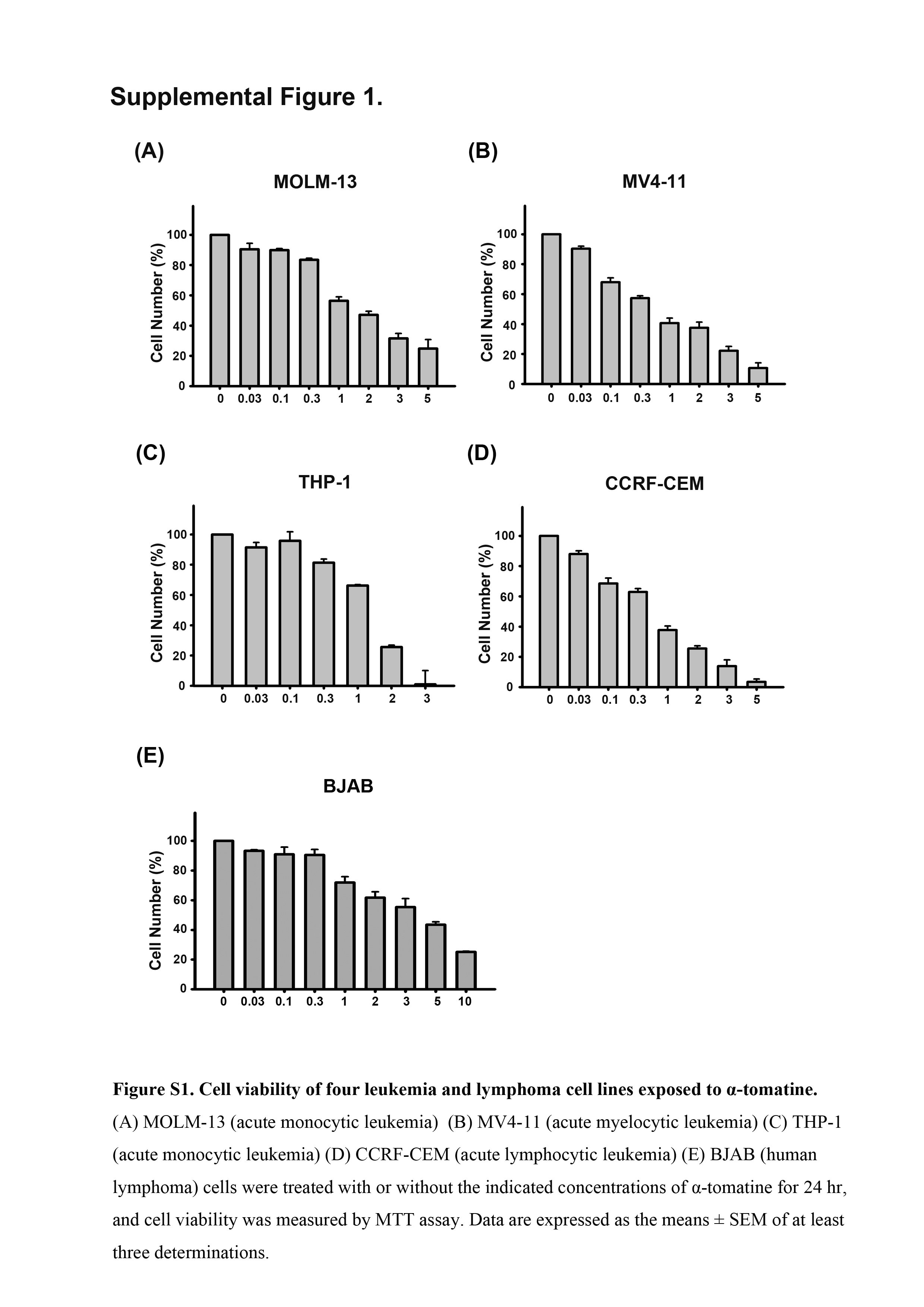

Supplement: Figure S1 — Cell viability of four leukemia and lymphoma cell lines exposed to α-tomatine. (A) MOLM-13 (acute monocytic leukemia) (B) MV4-11 (acute myelocytic leukemia) (C) THP-1 (acute monocytic leukemia) (D) CCRF-CEM (acute lymphepithelium) (E) BJAB (human lymphoma) cells were treated with or without treated the indicated concentrations of α-tomatine for 24 hr, and cell viability was measured by MTT assay. Data are expressed as the means ± SEM of at least three determinations. (TIF) [file pone.0044093.s001.tif]

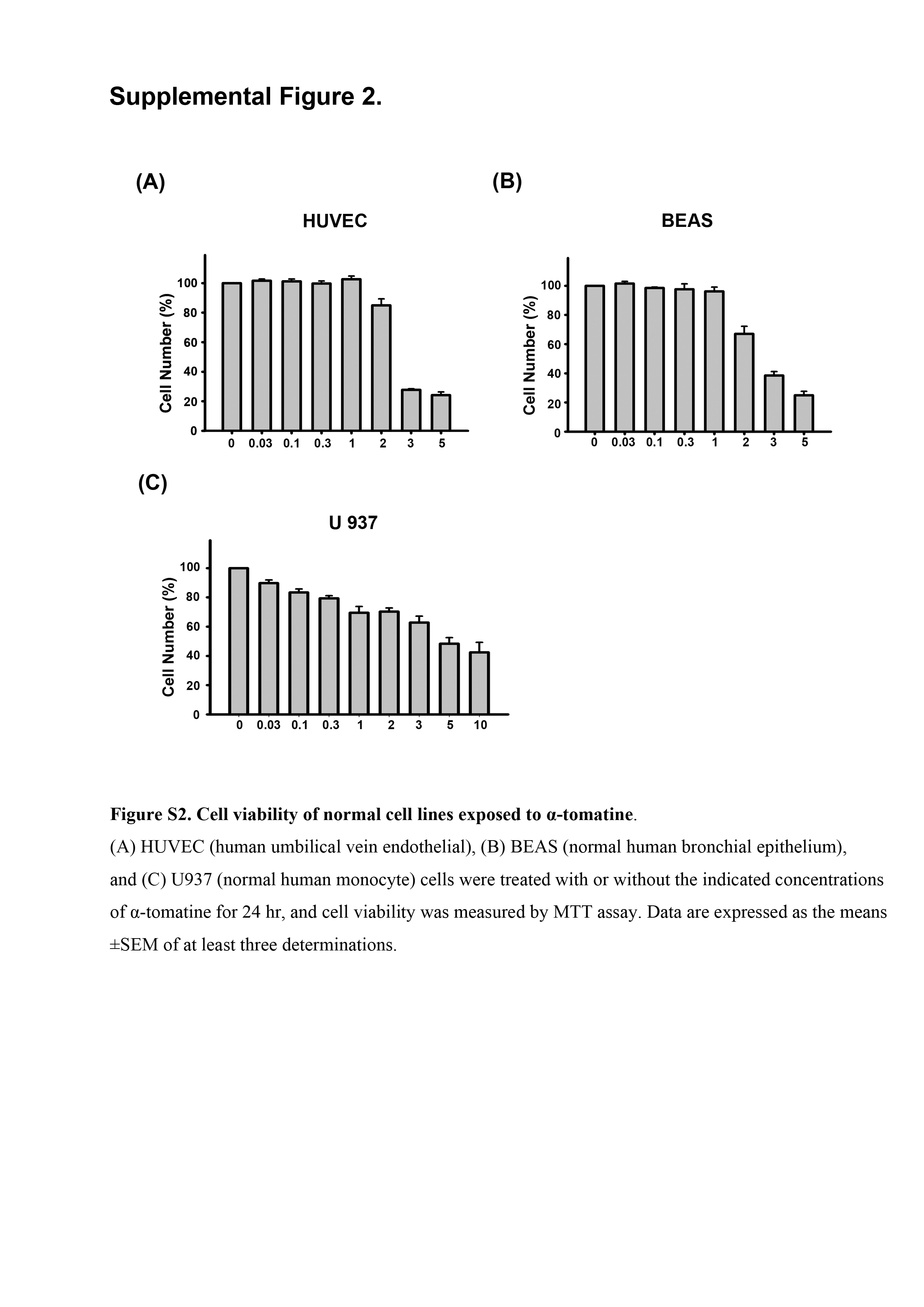

Supplement: Figure S2 — Cell viability of normal cell lines exposed to α-tomatine. (A) HUVEC (human umbilical vein endothelial), (B) BEAS (normal human bronchial epithelium), and (C) U937 (normal human monocyte) cells were treated with or without the indicated concentrations of α-tomatine for 24 hr, and cell viability was measured by MTT assay. Data are expressed as the means ± SEM of at least three determinations. (TIF) [file pone.0044093.s002.tif]

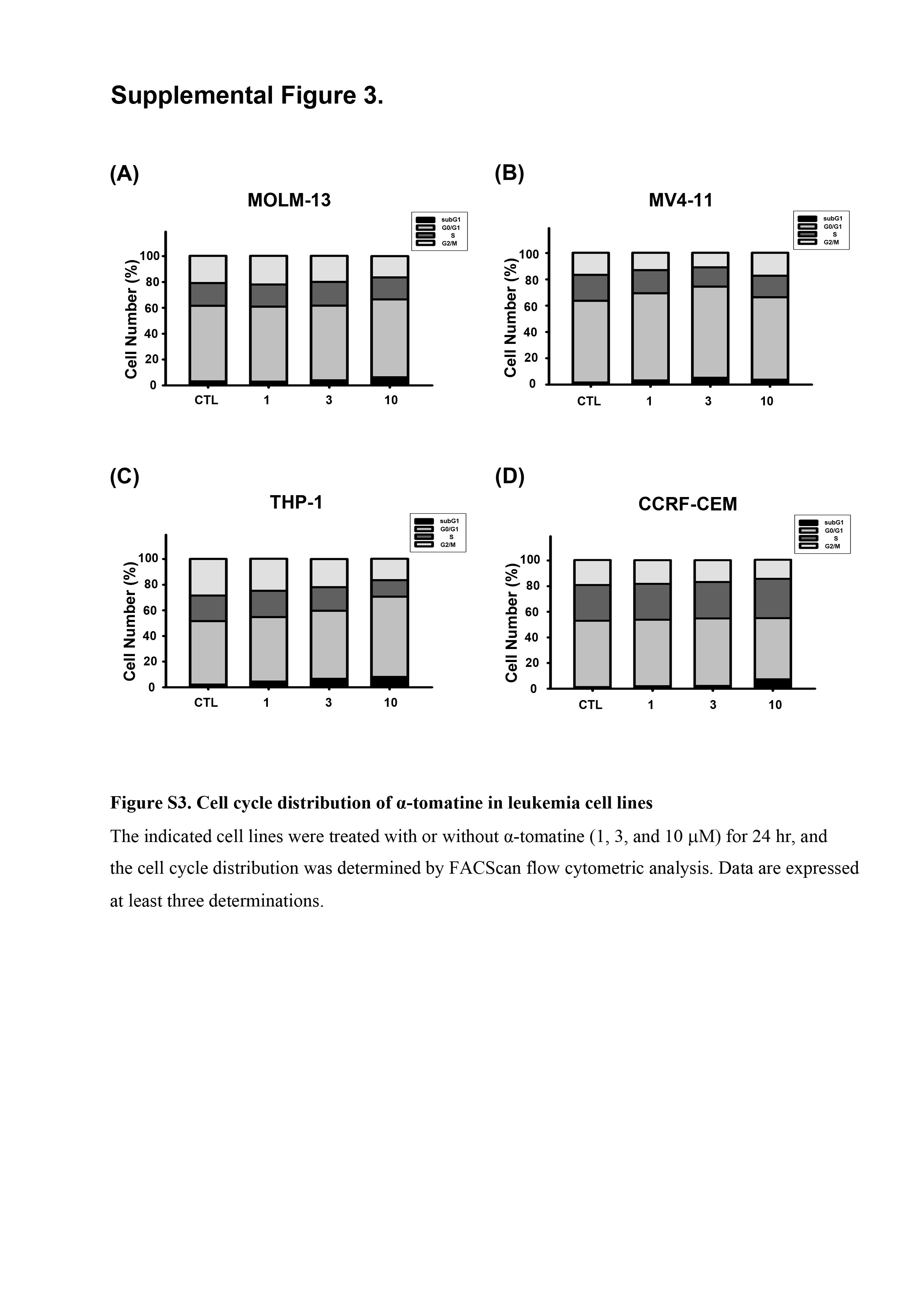

Supplement: Figure S3 — Cell cycle distribution of α-tomatine in leukemia cell lines. The indicated cell lines were treated with or without α-tomatine (1, 3, and 10 µM) for 24 hr, and the cell cycle distribution was determined by FACScan flow cytometric analysis. Data are expressed at least three determinations. (TIF) [file pone.0044093.s003.tif]

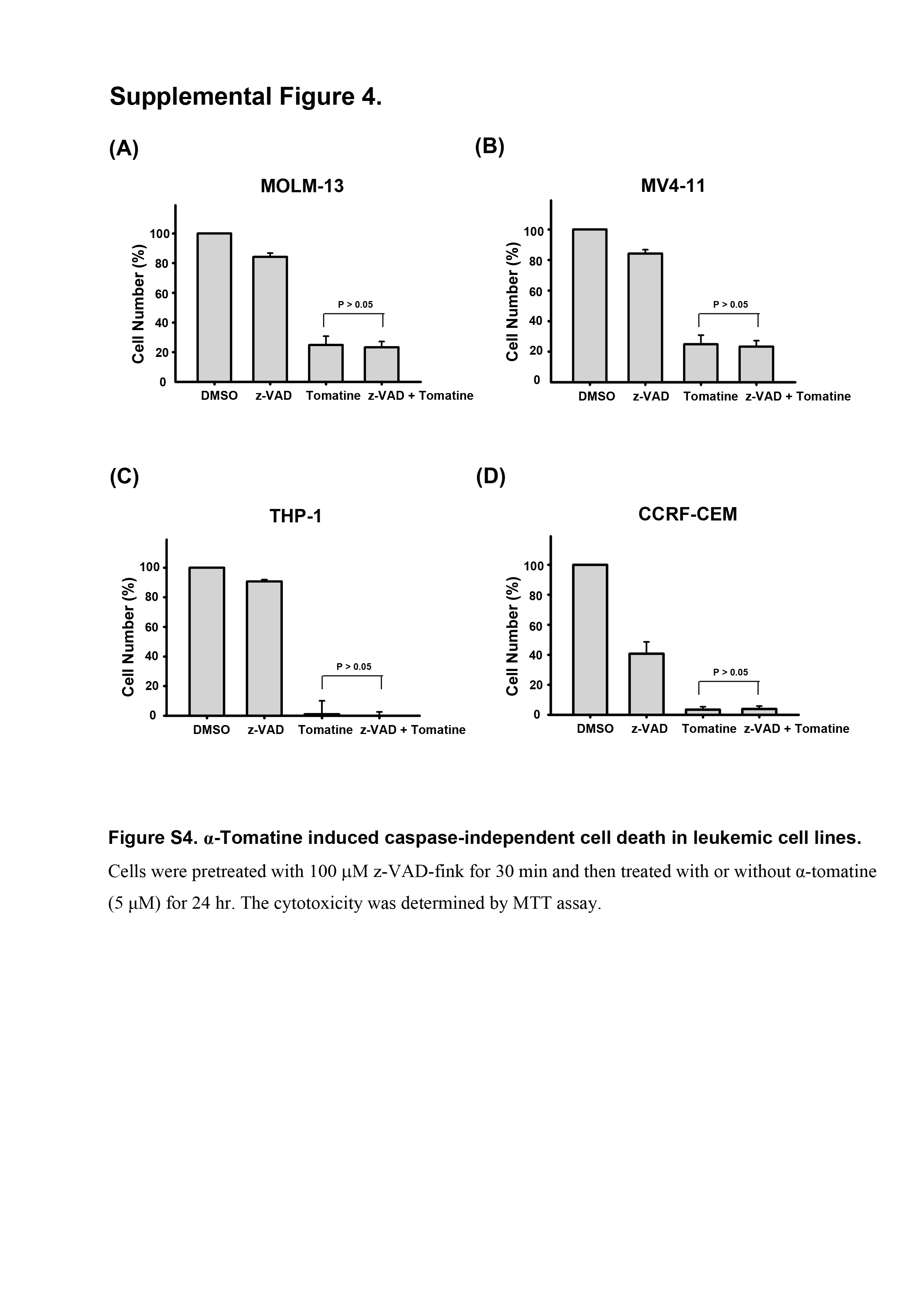

Supplement: Figure S4 — α-Tomatine induced caspase-independent cell death in leukemia cell lines. The indicated cells were pretreated with 100 µM z-VAD-fmk for 30 min and then treated with or without α-tomatine (5 µM) for 24 hr. The cytotoxicity was determined by MTT assay. (TIF) [file pone.0044093.s004.tif]

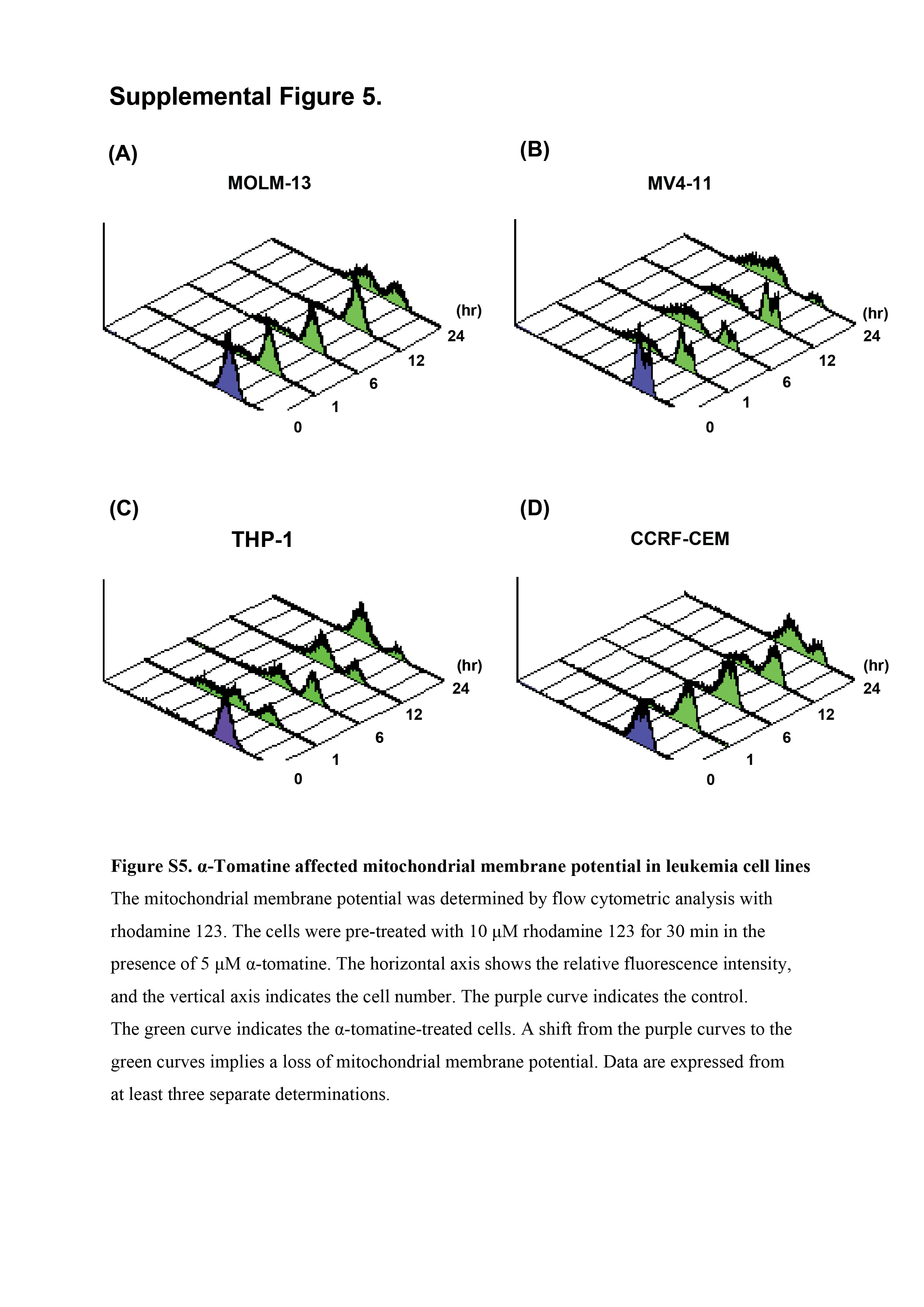

Supplement: Figure S5 — α-Tomatine affected mitochondrial membrane potential in leukemia cell lines. The mitochondrial membrane potential was determined by flow cytometric analysis with rhodamine 123. The cells were pre-treated with 10 µM rhodamine 123 for 30 min in the presence of 5 µM α-tomatine. The horizontal axis shows the relative fluorescence intensity, and the vertical axis indicates the cell number. The purple curve indicates the control. The green curve indicates the α-tomatine-treated cells. A shift from the purple curves to the green curves implies a loss of mitochondrial membrane potential. Data are expressed from at least three separate determinations. (TIF) [file pone.0044093.s005.tif]

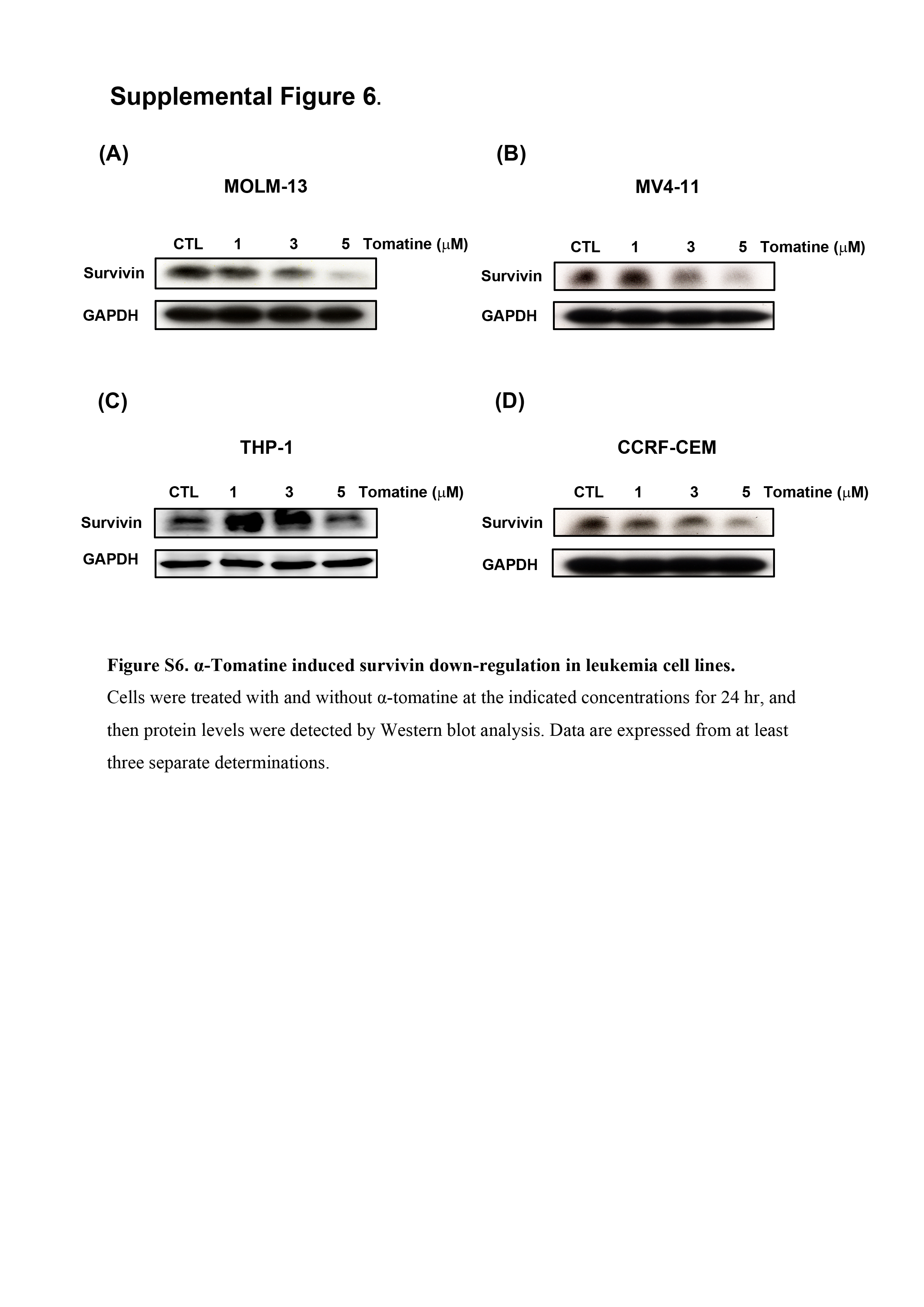

Supplement: Figure S6 — α-Tomatine induced survivin down-regulation in leukemia cell lines. Cells were treated with or without α-tomatine at the indicated concentrations for 24 hr, and then protein levels were detected by Western blot analysis. Data are expressed from at least three separate determinations. (TIF) [file pone.0044093.s006.tif]
